# Improving in-season wheat yield prediction using remote sensing and additional agronomic traits as predictors

**DOI:** 10.3389/fpls.2023.1063983

**Published:** 2023-04-03

**Authors:** Adrian Gracia-Romero, Rubén Rufo, David Gómez-Candón, José Miguel Soriano, Joaquim Bellvert, Venkata Rami Reddy Yannam, Davide Gulino, Marta S. Lopes

**Affiliations:** ^1^ Field Crops Program, Institute for Food and Agricultural Research and Technology (IRTA), Lleida, Spain; ^2^ Efficient Use of Water in Agriculture Program, Institute for Food and Agricultural Research and Technology (IRTA), Lleida, Spain

**Keywords:** wheat, grain yield, prediction models, UAV, NDVI, plant height, phenology

## Abstract

The development of accurate grain yield (GY) multivariate models using normalized difference vegetation index (NDVI) assessments obtained from aerial vehicles and additional agronomic traits is a promising option to assist, or even substitute, laborious agronomic in-field evaluations for wheat variety trials. This study proposed improved GY prediction models for wheat experimental trials. Calibration models were developed using all possible combinations of aerial NDVI, plant height, phenology, and ear density from experimental trials of three crop seasons. First, models were developed using 20, 50 and 100 plots in training sets and GY predictions were only moderately improved by increasing the size of the training set. Then, the best models predicting GY were defined in terms of the lowest Bayesian information criterion (BIC) and the inclusion of days to heading, ear density or plant height together with NDVI in most cases were better (lower BIC) than NDVI alone. This was particularly evident when NDVI saturates (with yields above 8 t ha^-1^) with models including NDVI and days to heading providing a 50% increase in the prediction accuracy and a 10% decrease in the root mean square error. These results showed an improvement of NDVI prediction models by the addition of other agronomic traits. Moreover, NDVI and additional agronomic traits were unreliable predictors of grain yield in wheat landraces and conventional yield quantification methods must be used in this case. Saturation and underestimation of productivity may be explained by differences in other yield components that NDVI alone cannot detect (e.g. differences in grain size and number).

## 1 Introduction

Wheat yield progress has been achieved at more than 1% *p. a.* in Europe and other parts of the world (Fischer et al., 2022; Lopes, 2022). Yield progress depends on direct experimental testing of novel agronomic practices and improved germplasm. Moreover, efficient research and innovation require modern, fast, accurate, and cost-effective tools to identify the most productive and sustainable wheat production strategies using large sets of experimental trials (several thousand plots) that can be readily transferred and adopted by producers as quickly as possible. For field evaluations, it is prevalent to find applications of high-throughput methodologies based on remote sensing; in particular, the use of unmanned aerial vehicles has become a popular topic for supporting crop breeding (Yang et al., 2017) owing to its high capacity for screening large populations rapidly and the moderate costs in comparison to traditional phenotyping procedures (Araus and Cairns, 2014). Among all the indices used, the versatility and simplicity of the normalized difference vegetation index (NDVI) across crop species (Gao et al., 2020; Tenreiro et al., 2021) and the possibility of measurement across a variety of platforms (Araus et al., 2021) have prompted the widespread use of NDVI for phenotyping purposes. However, even if a close relationship between grain yield and vegetation indices has been demonstrated under a wide range of growing conditions, these approximations are not considered universal solutions, as some limitations have been reported. Challenges are mainly attributed to the saturation effect during dense canopy assessment (Duan et al., 2017). In contrast to NDVI, LiDAR is not affected by saturation at high ground cover and might be an alternative for biomass (Jimenez-Berni et al., 2018); however, these models still have limitations in predicting grain yield, and alternatives are necessary to increase the accuracy and precision of vegetation indices.

Alternative models have been explored and reported in the literature using plant height (PH) together with NDVI in herbaceous crops, such as perennial ryegrass, to estimate biomass (Gebremedhin et al., 2019). Other candidate traits, such as phenology, may provide important information regarding how wheat genotypes perform in a given environment (Lafitte et al., 2003) and can assist in-season selection. The measurement of wheat PH (Rebetzke and Richards, 2000) and phenology (Lopes et al., 2018) helps in understanding the sensitivity of crop production to fluctuating seasonal conditions, as the duration of developmental phases is a key determinant of genetic adaptation to the environment. Among the wheat yield components, ear density per unit of ground area has been considered an important agronomic trait (Pask et al., 2012) that can be easily measured with image analysis (Fernandez-Gallego et al., 2018) and may improve the accuracy of yield prediction models. The development of new grain yield (GY) prediction models, including NDVI together with additional easy-to-measure agronomic traits, has the potential to address the NDVI saturation issues described, and eventually improve yield predictions. To explore this hypothesis, two case studies were used and carefully selected to demonstrate and investigate the mechanisms associated with NDVI saturation. The first case study consisted of a set of data obtained from landraces and modern varieties, whereas the second case study was characterized by trials under various agronomic testing conditions and a wide range of GY variation. For these two case studies, calibration curves or training sets were developed using various model combinations of GY, NDVI, and other easy-to-measure traits, including phenology, PH, and ear density (EARS), using a reduced number of plots. These calibrations were then used to predict the yield of the remaining plots (validation sets) and the correlations between the predicted and observed yields obtained for the various sets to select the best and most universal model.

## 2 Materials and methods

### 2.1 Site description, plant material, and experimental design

#### 2.1.1 Case study 1

Field experiments were conducted at an experimental station in Gimenells, Lleida, Spain 41°38′N, 00°22′E, 260 m a.s.l) in 2017 and 2018 under rainfed conditions. The environmental conditions of the study area are characterized by a temperate semi-arid climate with cool, wet winters, and dry and hot spring to summer seasons. The average annual precipitation is approximately 370 mm. The month with the lowest precipitation on average is July, with an average of 12.7 mm. The trials were sown on 21/11/2016 and 15/11/2017. In 2017 trial, after soil analysis, N, P and K were applied (pre-planting) to reach 50 kg of N/ha, 98 kg P/ha and 108 kg K/ha in the form of Calcium nitrate (NAC 27%), KCl and Ca(H_2_PO_4_)_2_. At tillering, 150 kg N/ha in the form of Calcium nitrate (NAC 27%) were additionally applied. In 2018 trial, N content in the soil was more than 200 kg/ha and only P and K were applied at the same rates used in 2017. The experiments followed a non-replicated augmented design with two replicated checks (‘Anza’ and ‘Soissons’) and plots of 3.6 m^2^ (1.2 m wide and 3 m long) with eight rows spaced 0.15 m apart. The seed rate was adjusted to 250 seeds per m^2^ and the plots were kept free of weeds and diseases. The germplasm assessed in Case Study 1 comprised 365 bread wheat (*Triticum aestivum* L.) genotypes from a diverse panel of landraces and modern wheat varieties (Rufo et al., 2019). This dataset obtained from landraces was of particular interest in this study to determine the limitations and challenges in predicting yield using the NDVI; Wheat landraces have high biomass (similar or even higher than that of modern wheat varieties), and consequently, high NDVI; however, this type of plant material has low GY and low harvest index, creating a bias towards yield predictions when using NDVI and additional agronomic traits (see **Supplementary Figure 1**). The GY ranges for each germplasm and the growing season are listed in **Table 1**.

**Table 1:** Grain yield (GY, t ha_-1_) means and standard deviation, number of plots, the minimum and maximum GY, and heritability (calculated only in replicated trials, H_2_) evaluated for each germplasm set, group of varieties, and growing conditions.

Case Study	Year	Exp.	Water treatment	Date of sowing	Gen.	N	Mean GY	Lowest GY	Highest GY	*H^2^*
1	2017	1	Rainfed	21/11/2016	354	Landrace, 170	5.10 ± 0.91	2.97	8.48	
						Modern, 184	9.48 ± 1.01	6.54	11.80	
	2018	1	Rainfed	15/11/2017	354	Landrace, 170	5.63 ± 0.82	3.65	8.99	
						Modern, 184	9.94 ± 0.98	6.93	12.40	
2	2021	1	Rainfed	27/12/2020	10	30	5.33 ± 1.61	1.87	9.00	0.688
		2	Irrigated	27/12/2020	10	30	8.87 ± 1.77	5.41	12.70	0.885
		3	Rainfed	03/12/2020	10	30	7.86 ± 2.17	3.77	11.83	0.776
		4	Irrigated	03/12/2020	10	30	10.32 ± 1.90	6.20	13.44	0.903
		5	Irrigated	03/12/2020	22	66	11.87 ± 1.54	8.05	14.64	0.678
		6	Irrigated	03/12/2020	22	66	10.55 ± 1.02	7.62	12.83	0.664
		7	Rainfed	03/12/2020	16	96	4.14 ± 1.09	2.17	7.18	0.697

#### 2.1.2 Case study 2

Field experiments were conducted at the experimental stations in Sucs, Lleida, Spain (41°38′N 00°22′E, 260 m a.s.l) in 2021, which is very close to the experimental station where Case Study 1 was conducted. A set of seven wheat experimental trials (with a total of 300 plots) conducted under rainfed and well-irrigated conditions with variable sowing dates and a diverse set of 39 modern wheat varieties were used to determine yield predictions. In all trials, after soil analysis, nitrogen contents in the soil were above 200 kg N/ha with no additional N requirements for optimal crop growth. Moreover, P and K were applied (pre-planting) to reach 98 kg P/ha and 108 kg K/ha with the same formulations used in case study 1. The experiments followed a replicated alpha-lattice design and plots of 9.6 m^2^ with eight rows spaced 0.15 m apart. The seed rate was adjusted to 250 seeds per m^2^, and the plots were kept free of weeds and diseases, as appropriate. This dataset is characterized by a wide range of GY variations retrieved from plots grown under various agronomic test conditions and sets of germplasm (all containing modern cultivated wheat varieties). This helped explore one of the limiting factors to NDVI prediction ability due to saturation. The GY ranges for each germplasm set and the growing season aspects are listed in **Table 1**.

### 2.2 Data acquisition and processing

In 2017 and 2018, remote sensing image acquisition was performed using a Parrot Sequoia multispectral camera onboard a hexacopter unmanned aerial vehicle. The Parrot Sequoia (Parrot, Paris, France) has a 1.2 mega-pixel sensor, yielding a resolution of 1280 × 960 pixels. The camera included four individual image sensors with filters centered at wavelengths and full-width half-maximum bandwidths (FWHM) of 550 ± 40 (green), 660 ± 40 (red), 735 ± 10 (red edge), and 790 ± 40 nm (near infrared). A Micasense RedEdge-M multispectral camera (Micasense, Seattle, Washington, USA) was used in 2021. This camera captured images at five spectral bands located at wavelengths of 475 ± 20 nm (blue), 560 ± 20 nm (green), 668 ± 10 nm (red), 717 ± 10 nm (red edge), and 840 ± 40 nm (near-infrared), and a field of view (FOV) of 47.2°. Image acquisition for all years was performed coinciding with the crop developmental stages of anthesis the 21/04/2017, 17/04/2018 and the 19/04/2021 (when more than 90% of the varieties reached anthesis). All flights were conducted at ~12:00 h solar time and at 40–50 m above ground level (agl), capturing images ground sampling distance of 50 m. The flight plan had an 80/60 frontal and side overlap. During image acquisition, *in situ* measurements were conducted for different targets to correct for atmospheric contributions to the signal. Radiometric calibration of the multispectral sensor was conducted using an external incident light sensor that measured the irradiance levels of light at the same bands as those of the camera. In addition to the radiometric corrections made by the internal solar irradiance sensor, corrections were conducted through *in situ* spectral measurements with black-and-white ground calibration targets, bare soil, and wheat plots using a JAZ-3 Ocean Optics STS VIS spectrometer (Ocean Optics, Inc., Dunedin, FL) with a wavelength response from 350 to 800 nm and an optical resolution of 0.3 to 10.0 nm. During spectral data collection, spectrometer calibration measurements were recorded with a reference panel (white color Spectralon™) and dark current before and after taking readings from the radiometric calibration targets. Geometric correction was conducted using ground control points. The position of each ground control point was acquired using a handheld global positioning system (Geo7x, Trimble GeoExplorer series, Sunnyvale, CA). All images were mosaicked using Agisoft Photoscan Professional version 1.6.2 (Agisoft LLC., St. Petersburg, Russia) software and geometric and radiometric terrain correction was performed using QGIS 3.4.15 (QGIS Development Team, Gossau, Switzerland). The NDVI values from each plot were calculated according to the equation shown below [1]:


[1] $$NDVI=\frac{\left(R790-R660\right)}{\left(R790+R660\right)}$$

The following agronomic traits were measured: phenology (days to heading, DH), plant height (PH), ear density (EARS), and GY (t ha^−1^). Days to heading was measured as the number of days between sowing and the day when 50% of spikes emerged in a plot (Zadoks Stage 59, Zadoks et al., 1974). Plant height was measured near maturity in 10 main stems per plot from the tillering node to the top of the spike, excluding the awns. The EARS was measured by counting the number of ears in one linear meter in the middle of each plot and calculating the number of ears per unit area (1 m^2^). Plots were mechanically harvested at ripening, and grain yield was calculated at 12% moisture.

### 2.3 Statistical analysis

Statistical analysis was performed using the open-source software R and RStudio 1.0.44 (R Foundation for Statistical Computing, Vienna, Austria), and all statistical analyses were equally applied in case studies 1 and 2. The strength of the relationships between the individual parameters DH, PH, EARS, NDVI and GY was examined using the Pearson correlation test. Broad sense heritability (*H^2^*) was estimated for each trait individually in each environment (only for replicated trials) as:


$$H^{2}=\frac{\sigma_{g}^{2}}{\left[\sigma_{g}^{2}+\left(\frac{\sigma^{2}}{r}\right)\right]}$$

where r=number of repetitions, σ^2^=error variance and σ^2^g =genotypic variance.

**Figure 1:**
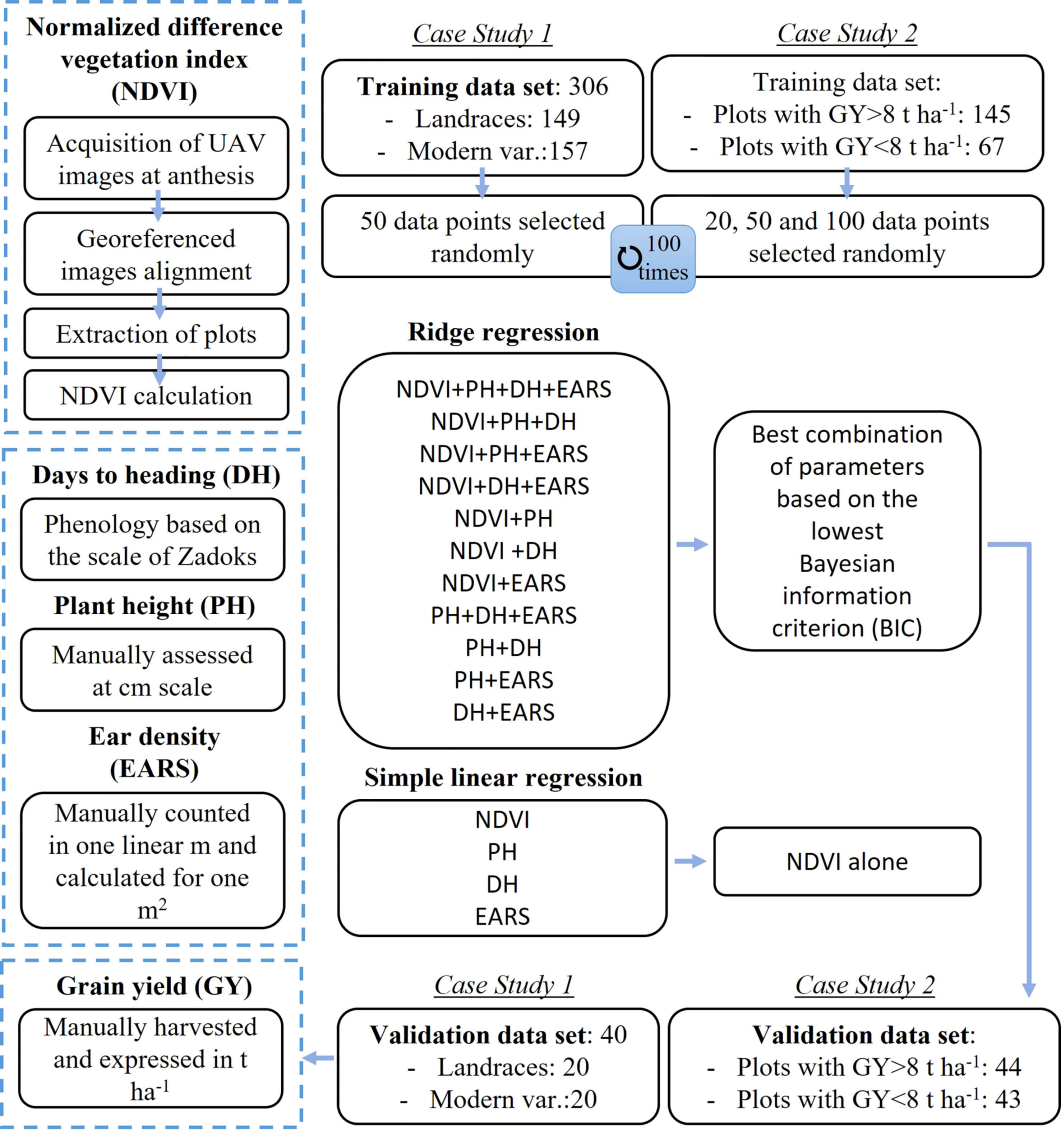
Flowchart of data acquisition and model elaboration. DH, days to heading; PH, plant height; NDVI, normalized difference vegetation index; EARS, ear density; UAV, unmanned aerial vehicle.

A multivariate approach was used to develop yield predicting models and procedures are illustrated in the flowchart shown in **Figure 1**. Multivariate ridge regression was selected as a model-tuning method to overcome multicollinearity among traits (Hoerl and Kennard, 2000). More complex models as Artificial Neural Networks were also considered, reporting very similar prediction accuracies (data not shown). However, we decided to perform the data analysis with Ridge Regression as is less likely to overfit the data and it provides a direct interpretation of feature importance. To perform the Ridge Regression, we used the functions from the glmnet package (Friedman et al., 2010). First, the lambda value that produces the lowest test mean squared error (MSE) was identified by k-fold cross validation using k = 10 folds.

### 2.4 Calibration of the yield prediction models

In order to find the best parameter combination, all possible 15 different models were developed to predict yield, including: (i) NDVI, PH, DH and EARS, (ii) NDVI, PH and DH, (iii) NDVI, PH and EARS, (iv) NDVI, DH and EARS, (v) NDVI and PH, (vi) NDVI and DH, (vii) NDVI and EARS, (viii) PH, DH and EARS, (ix) PH and DH, (x) PH and EARS, (xi) DH and EARS, (xii) NDVI, (xiii) PH, (xiv) DH and (xv) EARS.

First, data was split into training data sets, used to build the models, and validation data sets, not included in the training data set to evaluate model accuracy. In Case Study 1, for each of the two growing seasons evaluated, a total of 40 plots (20 landraces and 20 modern varieties) were randomly selected for the validation set. In Case Study 2, the validation data sets were comprised by the experimental conditions 2, 3 and 4 (**Table 1**); and the other two were used as two independent validations set: the experimental condition 1 (rainfed and late-planting) as low yielding plots and the experimental condition 5 (irrigation and normal planting) as high yielding plots (**Table 1**). For Case Study 1, multiple and simple regression models were constructed using 50 randomly selected plots from the training data sets, whereas for Case Study 2, models were constructed using 20, 50 and 100 randomly selected plots from the training data sets. For each model, 100 iterations were performed and, in each iteration, random plots were used to develop models. The best performing models were selected based on the lowest Bayesian information criterion (BIC) in each calibration subset. The best multiple regression model together with the best simple NDVI regression was used to directly predict yield of the validation data sets. The coefficients of determination (*R* ^2^), equation parameters, and associated probabilities were calculated for each yield multiple and simple regression models.

## 3 Results

### 3.1 Grain yield correlations with NDVI, PH, DH, and EARS

**Figure 2:**
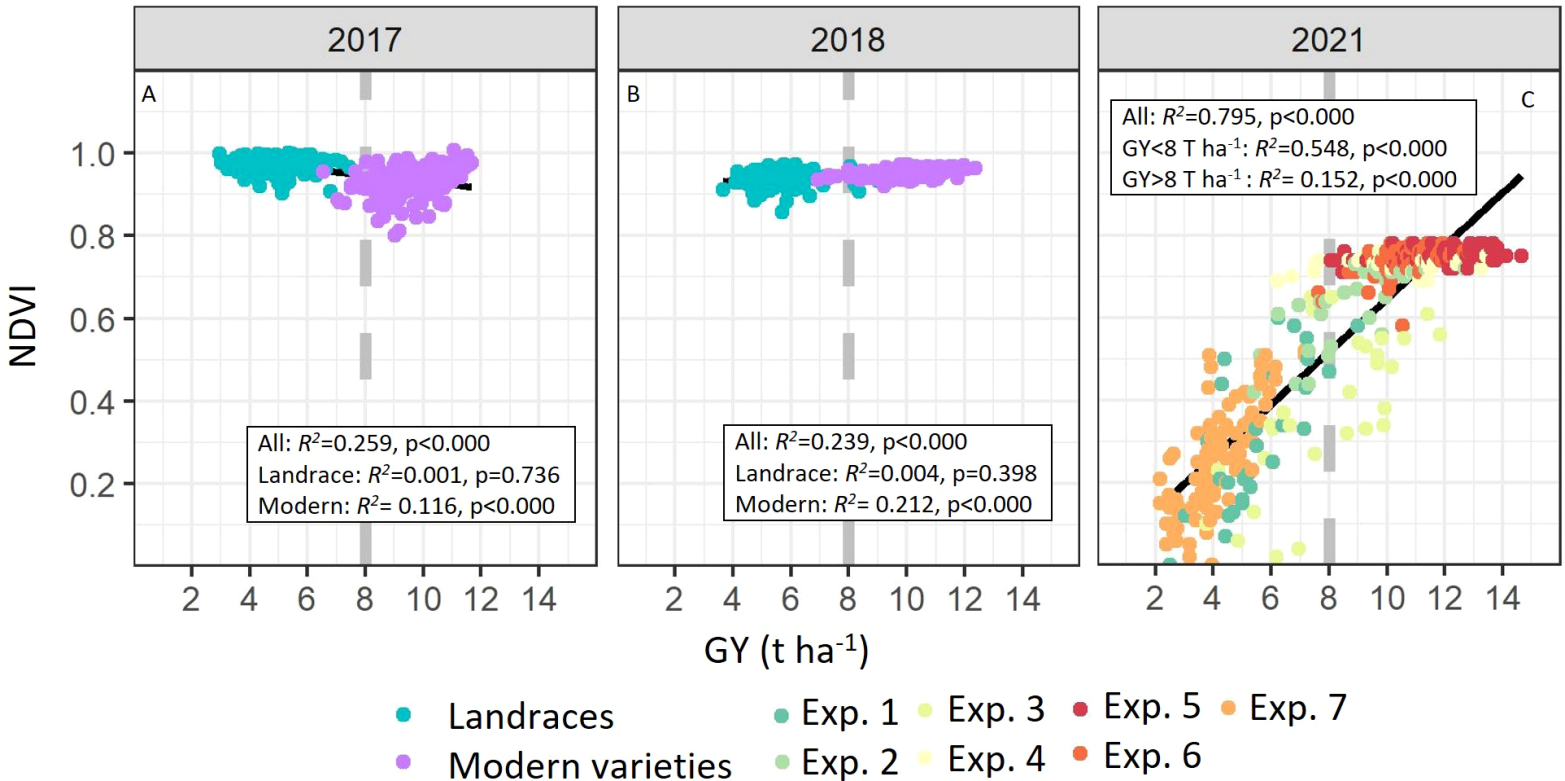
Linear relationships between grain yield (GY, t ha-1) with the normalized difference vegetation index (NDVI) measured at anthesis in Case Study 1 (**A**, 2017; **B**, 2018) and 2 (**C**, 2021). In case study 1 correlations were calculated separately in modern wheat varieties and landraces. In case study 2, correlations were also calculated separately in plots with yields below and above 8 t ha-1. Coefficients of determination (R2) and associated probabilities are shown. Dashed line represents the GY after which NDVI saturates. R2 within experimental conditions from case study 2 were 0.581 for Exp.1, 0.563 for Exp.2, 0.493 for Exp.3, 0.030 for Exp.4, 0.076 for Exp.5, 0.226 for Exp.6 and 0.549 for Exp.7.

To assess the correlation between NDVI and grain yield (GY), Pearson correlation coefficients were calculated (**Figure 2**). Significant correlations were reported across the complete set of plots (*R* ^2^ = 0.259, *R* ^2^ = 0.239, and *R* ^2^ = 0.795; *p<* 0.0001), for the 2017, 2018 and 2021 growing seasons, respectively. For Case Study 1, significant correlations were only reported for modern varieties (*R* ^2^ = 0.116, and *R* ^2^ = 0.212; *p<* 0.0001) but not in landraces. For Case Study 2, these correlations were also significant, however, NDVI saturated and did not change when plots showed yields above 8 t ha^-1^ (**Figure 2C**). When NDVI-GY correlation was tested for the two groups (below and above 8 t ha^-1^), regressions using data from plots with yields below 8 t ha^-1^ showed higher R^2^ (*R* ^2^ = 0.548; *p<* 0.0001) than regression obtained from plots with yields above 8 t ha^-1^ (*R* ^2^ = 0.152; *p<* 0.0001). To determine if yield prediction models would improve with the inclusion of additional agronomic traits when NDVI saturates, modelling and validations were calculated in the two groups of plots separately (below and above 8 t ha^-1^).

**Figure 3:**
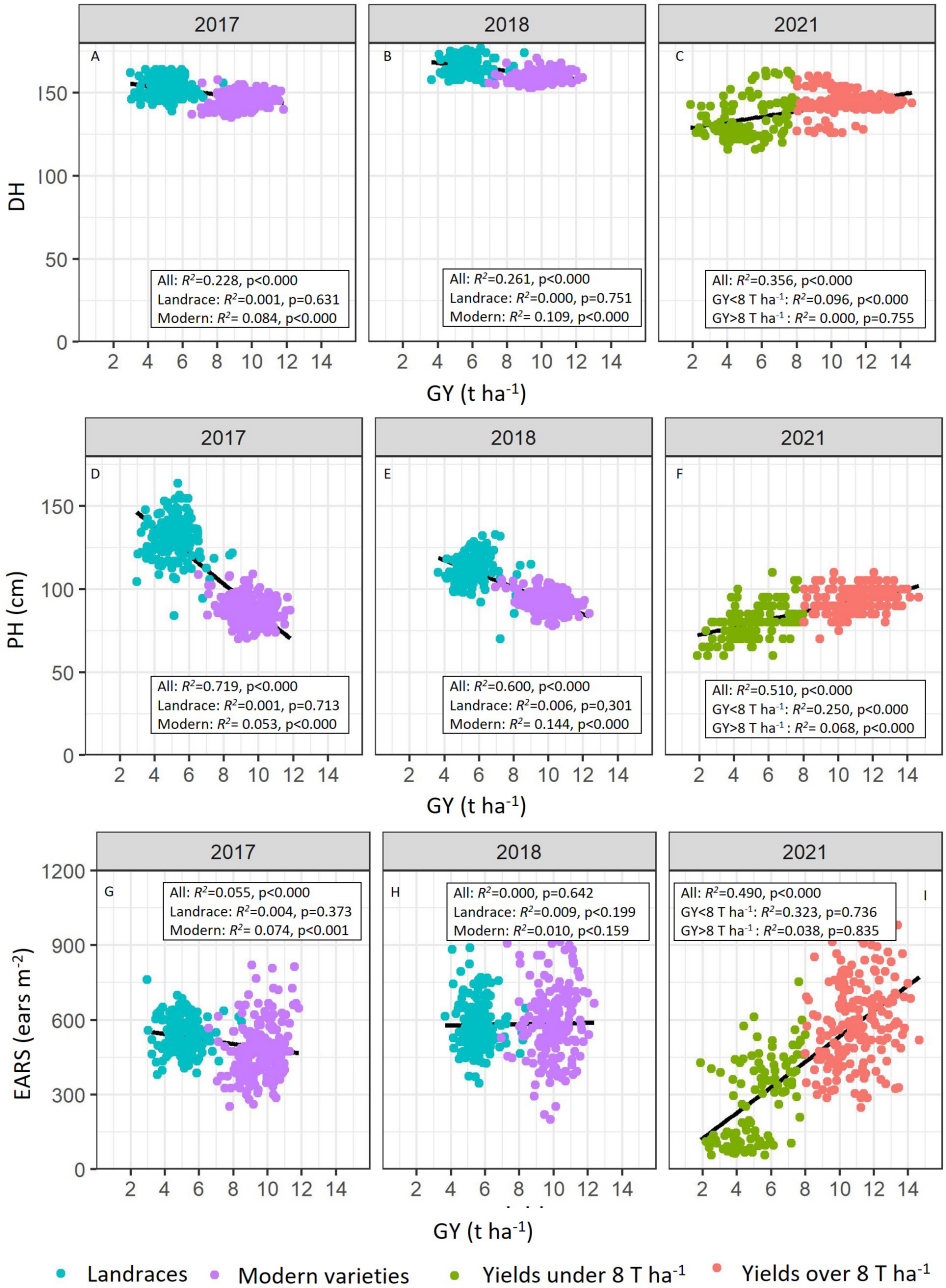
Linear relationships between grain yield (GY, t ha-1) and the number of days to heading (DH) (**A**, 2017; **B**, 2018; **C**, 2021), plant height (PH, cm) (**D**, 2017; **E**, 2018; **F**, 2021), and ear density (EARS, ears m-2) (**G**, 2017; **H**, 2018; **I**, 2021). Coefficients of determination (R2) and associated probabilities are shown.

Likewise, correlations between plant height (PH), phenology (DH), and ear density (EARS) and grain yield (GY) were calculated (**Figure 3**). Significant correlations were reported between DH–GY (*R* ^2^ = 0.228, *R* ^2^ = 0.261, and *R* ^2^ = 0.356; *p<* 0.0001), and PH–GY (*R* ^2^ = 0.719, *R* ^2^ = 0.600, and *R* ^2^ = 0.510; *p<* 0.0001) across the complete set of plots for the 2017, 2018, and 2021 growing seasons, respectively. The correlation between EARS and GY was also significant in 2017 (*R* ^2^ = 0.055, *p*< 0.0001) and in 2021 (*R* ^2^ = 0.49, *p*< 0.0001) (**Figure 3**).

### 3.2 Development and validation of simple and multiple regression models to predict grain yield

The objective of this step was to determine the minimum number of plots required for accurate grain-yield predictions. Data from Case Study 2 was used in this step and models were built within the groups set in the Results section 3.1 of plots yielding over and below 8 t ha^-1^ (threshold yield for NDVI saturation). For the models developed using plots with yields over 8 t ha^-1^, the best combination with the lowest Bayesian information criterion (BIC) was NDVI+DH (**Supplementary Table 1**). When models were trained using yields below 8 t ha^-1^, the best model was the combination of NDVI+PH+DH+EARS when 20 data points were used as training sets and the combination of NDVI+DH+EARS with the 50 data point training sets. In that case, *R^2^* was improved and the RMSE reduced as the training sets were increased (**Supplementary Table 1**).

### 3.3 Development and validation of models to predict grain yield in various wheat genetic resources (landraces and modern varieties): Case study 1

Prediction models using all possible trait combinations were constructed with data from Case Study 1 within landraces, within modern varieties and across the combination of both (**Supplementary table 2**). Yield prediction models obtained from modern varieties were significant, however in Landraces neither multiple nor single regressions were significant (**Supplementary Table 2**). The best yield prediction models obtained from modern wheat varieties with the lowest BIC included NDVI+DH (*R^2 =^* 0.24 and RMSE=0.86) in 2017 and NDVI in 2018 (*R^2 =^* 0.20 and RMSE=0.88). When GY predictions were modeled using both landraces and modern varieties, best model with the lowest BIC included single regression with PH (*R^2 =^* 0.71) in 2017 and multiple regression of NDVI+PH+EARS (*R^2 =^* 0.69) in 2018, reporting the highest model accuracies in terms of *R^2^* but the highest RMSE (RMSE=1.29 and RMSE=1.31, respectively).

**Figure 4:**
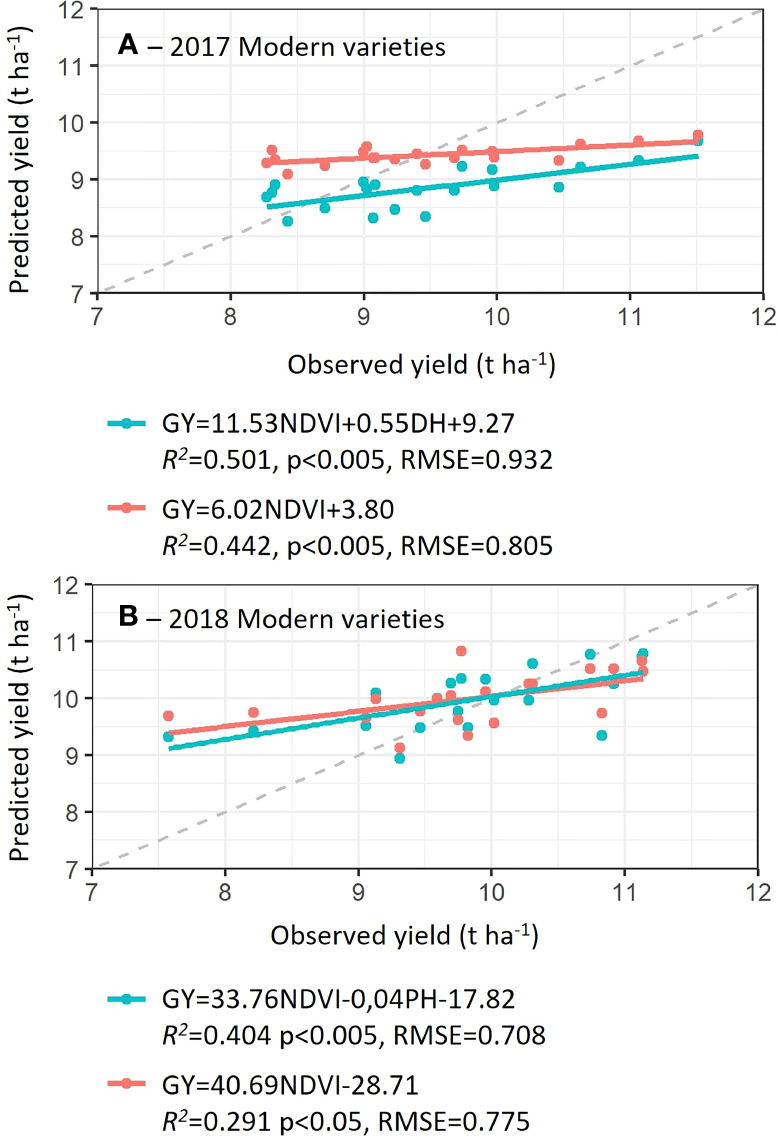
Prediction accuracies (coefficients of determination, *R^2^*), root means square root (RMSE) and probability of models developed with 50 data points from Case Study 1 using modern varieties in 2017 **(A)** and 2018 **(B)**. Predicted yield values were calculated in 20 plots not used in the development of models (irrigated and optimal sowing date). Dashed line indicates a 1:1 correlation.

Given the challenge of predicting landrace yields with the proposed parameters (with non- significant regressions), only models developed using modern varieties were validated. For the validation, the best model with the lowest BIC (using 50 data points) was selected and its accuracy to predict yield was compared with the accuracy of the NDVI simple regression (**Figure 4**), considered herein the benchmark model. For each validation, one model from all the 100 runs calculated was selected by sorting all the BIC values and selecting a model with the median BIC. For both growing seasons, the addition of agronomic parameters together with NDVI, to predict yield improved the prediction accuracies in comparison to simple NDVI models (**Figures 4A, B**).

### 3.4 Development and validation of models to predict grain yield of wheat variety testing trials with yield below and above 8 t ha^-1^: Case study 2

**Figure 5:**
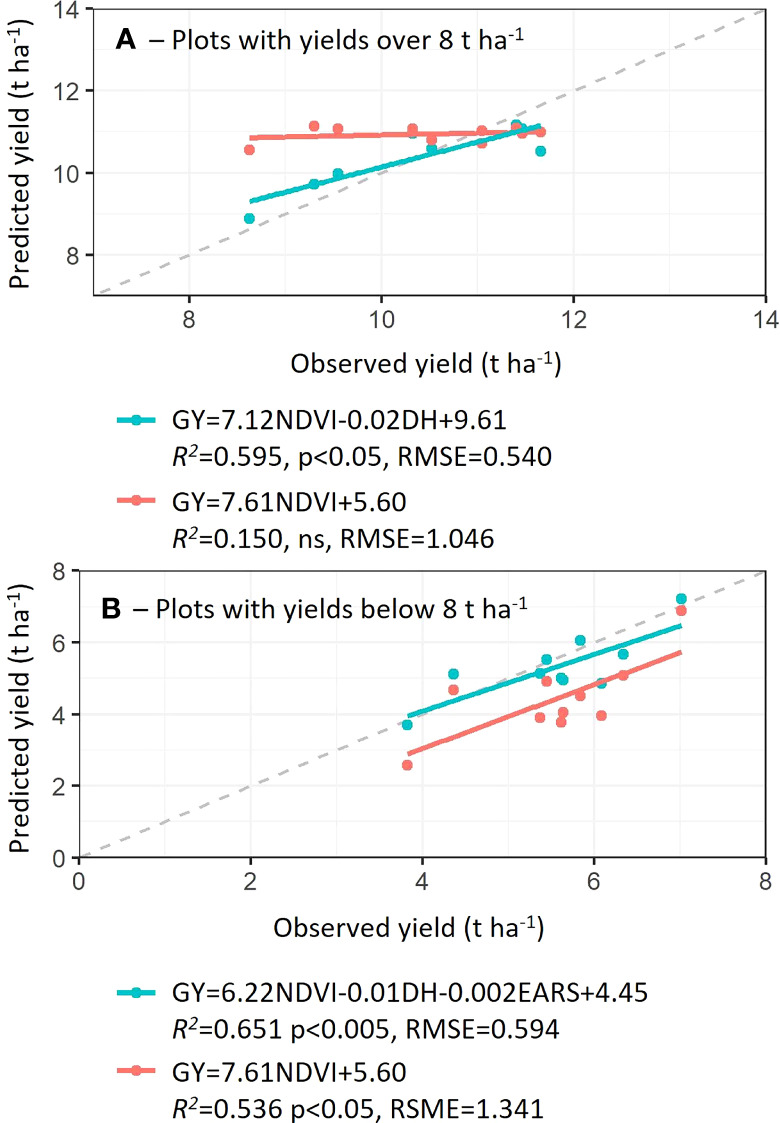
Prediction accuracies (coefficients of determination, *R^2^*), root means square root (RMSE) and probability of models developed with 50 data points from Case Study 2 using plots with yields over and below 8 t ha^-1^ **(A, B)**, respectively. Predicted yield values were calculated in 10 plots not used in the development of models (rainfed and late sowing). Dashed line indicates a 1:1 correlation.

Following the same procedure as in Case Study 1, best parameter combination was assessed to predict GY in the validation sets while comparing its accuracy with simple NDVI models (**Figure 5**). When plots with yields over 8 t ha^-1^ were evaluated, the model combining NDVI with DH significantly improved the yield prediction (*R^2 =^* 0.595, p<0.05) compared to the model using solely NDVI (*R^2 =^* 0.150, ns). For the selection of plots with yields under 8 t ha^-1^, even if the NDVI model reported a significant yield prediction (*R^2 =^* 0.536), the addition of DH and EARS improved yield predictions to *R^2 =^* 0.651 (**Figure 5**).

## 4 Discussion

### 4.1 Contributions of additional agronomic traits to improve remote sensing-based yield prediction models

Originally, NDVI was found to be an adequate indicator of plant biomass, chlorophyll content and N content (Stone et al., 1996; Babar et al., 2006; Tremblay et al., 2009; Hassan et al., 2019). Moreover, dynamic monitoring of NDVI in wheat trials to predict yield was later confirmed by direct correlations between NDVI and yield particularly at anthesis (Duan et al., 2017; Goodwin et al., 2018). Biomass, chlorophyll and Nitrogen content are physiological components of yield, however, biomass partition to yield may vary and higher biomass, chlorophyll and N may not result in higher yields. Herein, two case studies were used to determine the accuracy and reliability of the yield prediction ability of NDVI with agronomic traits such as DH, PH, and EARS. Overall, in both case studies, NDVI was, at least in plots with yields below 8 T ha^-1^ and using cultivated modern wheat varieties, an adequate predictor of yield (with prediction accuracies of up to *R^2 =^* 0.536; **Figure 5B**). The addition of agronomic traits such as DH to NDVI, in multiple regression models to predict yield improved prediction accuracies by up to 75% in plots with yields above 8 T ha^-1^ as compared to simple regression NDVI models. However, the accuracy obtained from multiple regression models (NDVI+DH+EARS was the best model with lowest BIC) to predict yields below 8 t ha^-1^ was 18% higher than simple regression models using NDVI. These results, support the hypothesis that the addition of simple to measure additional agronomic traits to NDVI in yield prediction models increased prediction acuracy. Moreover, phenology, i.e. (days to heading, DH), plant height (PH), ear density (EARS) are agronomic traits which all have the potential to be measured non-destructively in high throughput using proximal and aerial sensing devices. Potentially, in the future, yields will be accurately predicted using functions that model contributions of these various traits reducing harvest costs of breeding programs.

### 4.2 Mechanisms of NDVI saturation and underestimation of productivity

The assumptions of a linear relationship between GY and NDVI are not always fulfilled because of the reduced sensitivity of this vegetation index to large biomass (Huete et al., 1985). One of the most prominent and discussed limitations of remote-sensing-based studies is the saturation found with dense canopies, which underestimates productivity (Chen et al., 2006; Gu et al., 2013). Herein, saturation at high NDVI values is clearly demonstrated in case studies 1 and 2. In Case Study 1, yield in wheat landraces was weakly correlated with NDVI and additional easy-to-measure agronomic traits, such as DH, PH, and EARS. Moreover, yield prediction models using these traits in landraces were never adequate showing non-significant R^2^. Compared to semi-dwarf cultivars with a high harvest index, landraces show relatively high biomass and high or similar NDVI (see **Figures 2A, B**), whereas yields and harvest index are low (Jaradat, 2013; Lopes et al., 2015 and **Supplementary Figure 1**) resulting in poor correlation and prediction ability. It has been previously reported that reductions in plant height and biomass associated with the Rht-B1b (formerly Rht1) and Rht-D1b (formerly Rht2) alleles in modern varieties increased grain yield, spike dry matter, grains m^−2^ and harvest index (Gale and Youssefian, 1985; Flintham et al., 1997) at the expense of stem dry matter (Fischer, 1985). The mechanisms underlying this trade-off are yet to be discovered, however, the results observed by Fischer (1985) and Flintham et al. (1997) support our observations that biomass in tall landraces (and high NDVI) is increased at the expense of yield loss. As such, higher biomass and NDVI in the landraces did not result in higher yields in this set of germplasm. It is concluded that the yield of landraces must be assessed directly and traditionally harvested and weighted due to a lack of yield prediction accuracy from models developed with NDVI and additional agronomic traits.

Further evidence of productivity saturation underestimation was observed in Case Study 2, where NDVI and yield prediction models were less robust, at yields above 8 t ha^-1^ and NDVI above 0.75. This can be explained by differences in grain yield components in high-yielding plots, including grain number and size (Sukumaran et al., 2018) which NDVI alone cannot detect. However, when DH was included in the prediction models, the accuracies were considerably improved (to *R^2 =^* 0.595). These results highlight the importance of developing new and more sensitive indices (Huete et al., 2002; Gracia-Romero et al., 2019) to improve performance predictions under high-yielding conditions together with the inclusion of easy to measure additional agronomic traits in prediction models.

### 4.3 Can NDVI measurements replace machine-harvested and seed-weighted yield determination in experimental wheat field trials?

The development of accurate yield prediction models is of key importance to facilitate the adoption of new wheat varieties and best agronomic practices. If sufficiently solid algorithms with reduced error in assessing GY are achieved, it might be possible to avoid the harvest of the whole panel of experimental plots, reducing the costs and efforts of the selection process. The actual replacement of labor-intensive harvested yields determined by machine harvest and seed weight in the field with yields predicted from NDVI and agronomic trait based models would be particularly useful for multi-location trials where seed recovery is not essential. Most countries worldwide perform regional evaluations of value for cultivation and use testing, and these networks would benefit from accurate yield prediction models.

The methodology proposed in this study suggests using a reduced number of wheat plots in experimental field trials to calibrate an optimized model to predict the yield of the remaining plots. A similar evaluation of the calibration and training size was presented by Tehseen et al. (2021), who demonstrated the effect of different population sizes of landraces in developing genome prediction methods and assisting the selection of rust-resistant wheat genotypes. Herein, the larger the training sets were, the more robust the models were, however, mean accuracies were very similar among the dataset sizes evaluated as the loss of predictive accuracy was reasonably small when the number of replicates sampled for the training set was reduced to 20 in comparison to the sets with 100 plots. Thus, following a plot selection criterion based on NDVI and additional agronomic traits, could help reduce the number of field plots to be machine harvested for calibration of the model. Moreover, to avoid NDVI saturation at high yielding growth conditions, calibration models must be developed separately according to data obtained from different treatments either with optimal crop management or with yield limiting factors (e.g. drought, heat or others) requiring separate model training.

To date, many studies have used different empirical models developed using NDVI to successfully predict wheat GY. However, most of the highest predictions are based on using accumulated NDVI values across crop development stages and collecting data across different years (as Aranguren et al. (2020) with *R* ^2^ = 0.89 and n = 204) or combining information from different study sites and using satellite information (as Lopresti et al. (2015) with *R* ^2^ = 0.56 and n = 90). In similar evaluations (data across a single growing season and from a unique experimental field) when GY differences are evaluated among genotypes grown under irrigated (i.e., high-yielding conditions), prediction accuracies are limited (as Naser et al. (2020) r = 0.47 and n = 72). Given the reported improvements achieved with the addition of DH, PH, and EARS to the models, opportunities to find proxies capable of evaluating those parameters directly from NDVI and high-throughput platforms will help to better select varieties in a cost-effective manner.

## 5 Conclusions

The proposed models combining NDVI with additional agronomic traits improved GY prediction of wheat varieties compared to models using NDVI as the sole predictor. These demonstrations will benefit the application of remote sensing in breeding programs, thereby providing more confidence in the selection of varieties using proxies. Remote sensing-based models showed a high potential to discriminate between wheat genotypes within a field, but only at GY lower than 8 t·ha^-1^, after which the GY prediction models were less robust. Similarly, the accuracy was reduced when landraces were assessed. Accuracy reduction was associated with NDVI saturation owing to (i) high biomass and low harvest index in landraces and (ii) under high yielding conditions when wheat varieties share high biomass but differ in other yield components (grain size and number). Therefore, using conventional harvest is advisable when testing landraces and adaptation to yield potential conditions (high yield with optimal agronomic management), at least until novel or improved models are available.

## Data availability statement

The raw data supporting the conclusions of this article will be made available by the authors, without undue reservation.

## Author contributions

AG-R analyzed the data and wrote the manuscript. RR collected and processed the data. DG-C collected and processed the data and reviewed and edited the manuscript. JS reviewed and edited the manuscript. JB collected and processed the data and reviewed and edited the manuscript. VRRY reviewed and edited the manuscript. DG reviewed and edited the manuscript. MSL designed the study and prepared the manuscript. All authors contributed to the article and approved the submitted version.
