# Supplementary material for: Improving in-season wheat yield prediction using remote sensing and additional agronomic traits as predictors
Source: Front Plant Sci. 2023 Apr 3;14:1063983. doi: 10.3389/fpls.2023.1063983 (PMC10106603; doi:10.3389/fpls.2023.1063983)
Supplement: Supplementary file 1 [file DataSheet_1.docx]

**
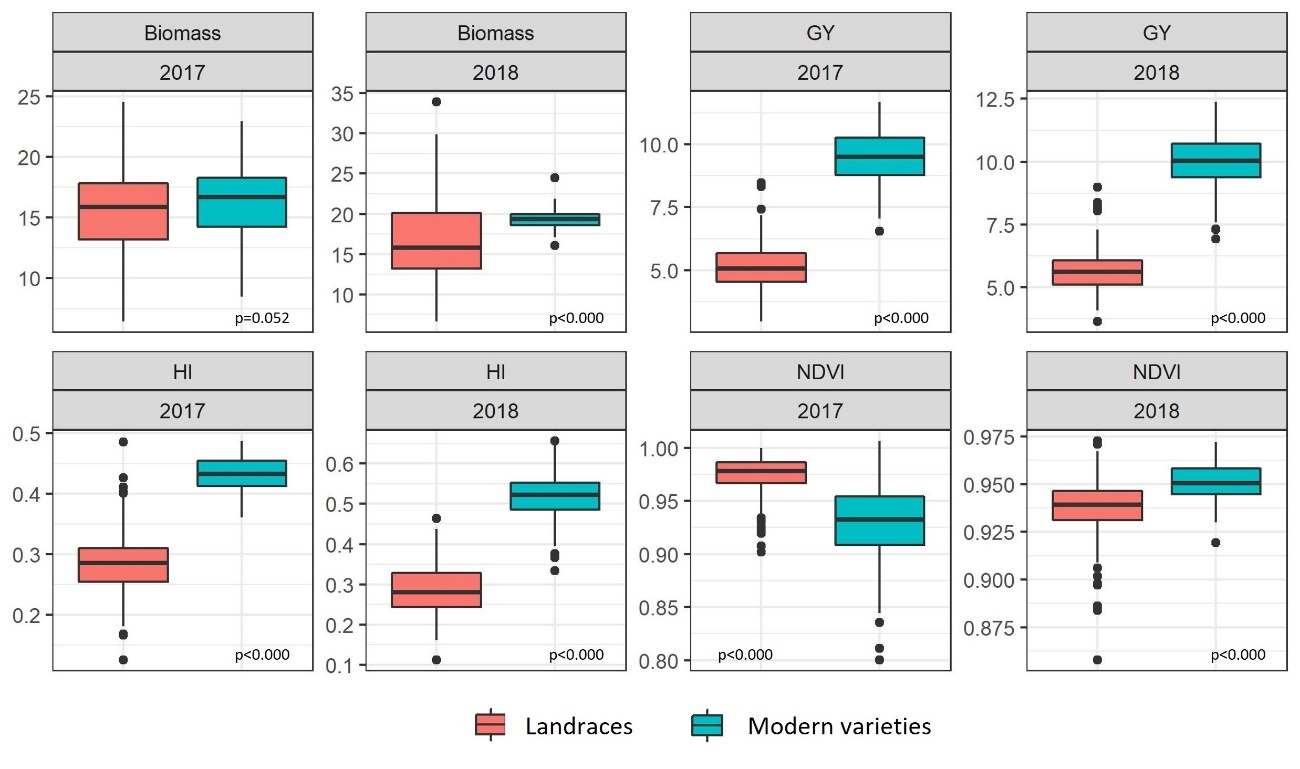
**

**Supplemental figure 1**. Boxplot of biomass (t ha^-1^), grain yield (GY, t ha^-1^), harvest index (HI) and NDVI showing the differences between the type of plant material (landraces and modern varieties) used in the Case Study 1. Statistical analyses were carried out by a one-way ANOVA and significant differences were determined at p-value ≥ 0.05.

**Supplemental table 1**. Parameters of multiple and simple regression models to predict grain yield (GY) from Case Study 2. Values are means of 100 runs developed for each model. Models were constructed with 20, 50, and 100 (only for plots with GY over 8 t ha^-1^) randomly selected from the training sets. The number of data points for plots with GY below 8 T ha^-1^ was lower and training models could only be developed with 20 and 50 plots. Models are sorted by Bayesian information criterion (BIC) from lowest to highest. NDVI, normalized difference vegetation index; DH, days to heading; PH, plant heigh; EARS, ears density; R^2^, coefficient of determination; RSME, root square mean error.

|  | **GY over 8 t ha^-1^** | | | |
| --- | --- | --- | --- | --- |
|  |  |  |  |  |
| **Training** | **Model** | ***R^2^*** | **RSME** | **BIC** |
|  |  |  |  |  |
| 20 | *NDVI, DH* | 0.26 | 1.22 | 72.83 |
|  | *NDVI* | 0.15 | 1.31 | 73.84 |
|  | *NDVI, PH, DH* | 0.26 | 1.21 | 74.35 |
|  | *NDVI, DH, EARS* | 0.25 | 1.22 | 74.68 |
|  | *NDVI, PH* | 0.16 | 1.30 | 75.36 |
|  | *NDVI, EARS* | 0.15 | 1.31 | 75.71 |
|  | *PH* | 0.07 | 1.37 | 75.82 |
|  | *NDVI, PH, DH, EARS* | 0.26 | 1.21 | 76.08 |
|  | *EARS* | 0.05 | 1.39 | 76.32 |
|  | *DH* | 0.02 | 1.41 | 76.89 |
|  | *PH, EARS* | 0.09 | 1.35 | 76.93 |
|  | *NDVI, PH, EARS* | 0.16 | 1.30 | 77.04 |
|  | *PH, DH* | 0.08 | 1.36 | 77.25 |
|  | *DH, EARS* | 0.05 | 1.38 | 77.94 |
|  | *PH, DH, EARS* | 0.11 | 1.34 | 78.38 |
|  |  |  |  |  |
| 50 | *NDVI, DH* | 0.25 | 1.24 | 175.89 |
|  | *NDVI, PH, DH* | 0.26 | 1.24 | 178.48 |
|  | *NDVI, DH, EARS* | 0.25 | 1.25 | 178.96 |
|  | *NDVI* | 0.15 | 1.33 | 179.77 |
|  | *NDVI, PH, DH, EARS* | 0.26 | 1.24 | 181.29 |
|  | *NDVI, PH* | 0.16 | 1.32 | 181.89 |
|  | *NDVI, EARS* | 0.15 | 1.33 | 182.76 |
|  | *PH* | 0.06 | 1.40 | 184.68 |
|  | *NDVI, PH, EARS* | 0.16 | 1.32 | 184.81 |
|  | *EARS* | 0.04 | 1.41 | 185.96 |
|  | *PH, EARS* | 0.09 | 1.38 | 186.20 |
|  | *PH, DH* | 0.06 | 1.40 | 187.58 |
|  | *DH* | 0.00 | 1.44 | 188.06 |
|  | *DH, EARS* | 0.03 | 1.42 | 188.98 |
|  | *PH, DH, EARS* | 0.09 | 1.38 | 188.98 |
|  |  |  |  |  |
| 100 | *NDVI, DH* | 0.25 | 1.24 | 342.48 |
|  | *NDVI, PH, DH* | 0.26 | 1.24 | 344.95 |
|  | *NDVI, DH, EARS* | 0.25 | 1.25 | 346.50 |
|  | *NDVI, PH, DH, EARS* | 0.26 | 1.24 | 349.05 |
|  | *NDVI* | 0.15 | 1.33 | 351.86 |
|  | *NDVI, PH* | 0.16 | 1.32 | 354.10 |
|  | *NDVI, EARS* | 0.15 | 1.32 | 355.27 |
|  | *NDVI, PH, EARS* | 0.16 | 1.32 | 357.70 |
|  | *PH* | 0.06 | 1.40 | 362.17 |
|  | *PH, EARS* | 0.09 | 1.38 | 362.77 |
|  | *EARS* | 0.04 | 1.41 | 363.93 |
|  | *PH, DH* | 0.06 | 1.40 | 365.78 |
|  | *PH, DH, EARS* | 0.09 | 1.37 | 366.03 |
|  | *DH, EARS* | 0.04 | 1.41 | 367.69 |
|  | *DH* | 0.00 | 1.44 | 368.56 |
|  |  |  |  |  |

|  | **GY below 8 T ha^-1^** | | | |
| --- | --- | --- | --- | --- |
|  |  |  |  |  |
| **Training** | **Model** | ***R^2^*** | **RSME** | **BIC** |
|  |  |  |  |  |
| 20 | NDVI, PH, DH, EARS | 0.73 | 0.77 | 58.01 |
|  | NDVI, DH, EARS | 0.69 | 0.83 | 59.09 |
|  | NDVI, EARS | 0.67 | 0.87 | 59.09 |
|  | NDVI, PH, EARS | 0.68 | 0.85 | 60.02 |
|  | NDVI | 0.55 | 1.00 | 63.30 |
|  | NDVI, PH | 0.58 | 0.98 | 64.20 |
|  | NDVI, DH | 0.54 | 1.01 | 65.58 |
|  | NDVI, PH, DH | 0.57 | 0.99 | 66.33 |
|  | PH, EARS | 0.44 | 1.14 | 70.39 |
|  | PH, DH, EARS | 0.46 | 1.12 | 71.26 |
|  | EARS | 0.30 | 1.27 | 72.83 |
|  | DH, EARS | 0.30 | 1.28 | 74.84 |
|  | PH | 0.21 | 1.36 | 75.69 |
|  | PH, DH | 0.22 | 1.35 | 77.33 |
|  | DH | 0.09 | 1.47 | 78.79 |
|  |  |  |  |  |
| 50 | NDVI, DH, EARS | 0.85 | 0.64 | 113.15 |
|  | NDVI, PH, DH, EARS | 0.85 | 0.64 | 116.08 |
|  | NDVI, EARS | 0.83 | 0.69 | 117.74 |
|  | NDVI | 0.81 | 0.73 | 120.36 |
|  | NDVI, PH, EARS | 0.83 | 0.70 | 121.43 |
|  | NDVI, DH | 0.81 | 0.73 | 123.02 |
|  | NDVI, PH | 0.81 | 0.74 | 123.55 |
|  | NDVI, PH, DH | 0.81 | 0.73 | 126.27 |
|  | PH, EARS | 0.68 | 0.94 | 148.55 |
|  | PH, DH, EARS | 0.70 | 0.92 | 149.20 |
|  | EARS | 0.58 | 1.09 | 160.25 |
|  | DH, EARS | 0.57 | 1.10 | 163.61 |
|  | PH, DH | 0.42 | 1.28 | 178.79 |
|  | DH | 0.33 | 1.37 | 183.22 |
|  | PH | 0.30 | 1.40 | 185.33 |
|  |  |  |  |  |

**Supplemental table 2.** Multiple and simple regression models to predict grain yield (GY) from Case Study 1. Values are means of 100 runs developed for each model. Models were constructed with 50 randomly selected plots from the training sets. Models are sorted by Bayesian information criterion (BIC) from lowest to highest. NDVI, normalized difference vegetation index; DH, days to heading; PH, plant heigh; EARS, ears density; R^2^, coefficient of determination; RSME, root square mean error.

|  |  | **2017** | | | |
| --- | --- | --- | --- | --- | --- |
|  |  |  |  |  |  |
| **Set** |  | **Parameters** | ***R^2^*** | **RSME** | **BIC** |
|  |  |  |  |  |  |
| **Landraces** |  | *EARS* | 0.01 | 142.90 | 0.92 |
|  |  | *DH* | 0.00 | 143.19 | 0.92 |
|  |  | *PH* | -0.01 | 143.26 | 0.93 |
|  |  | *NDVI* | -0.01 | 143.36 | 0.93 |
|  |  | *DH, EARS* | 0.00 | 145.97 | 0.92 |
|  |  | *NDVI, EARS* | -0.01 | 146.06 | 0.93 |
|  |  | *PH, EARS* | -0.01 | 146.11 | 0.93 |
|  |  | *PH, DH* | -0.01 | 146.35 | 0.93 |
|  |  | *NDVI, PH* | -0.01 | 146.47 | 0.93 |
|  |  | *NDVI, DH* | -0.01 | 146.47 | 0.93 |
|  |  | *PH, DH, EARS* | 0.00 | 148.95 | 0.92 |
|  |  | *NDVI, DH, EARS* | -0.01 | 149.17 | 0.93 |
|  |  | *NDVI, PH, EARS* | -0.01 | 149.22 | 0.93 |
|  |  | *NDVI, PH, DH* | -0.02 | 149.82 | 0.93 |
|  |  | *NDVI, PH, DH, EARS* | -0.02 | 152.32 | 0.93 |
|  |  |  |  |  |  |
| **Modern** |  | *NDVI, DH* | 0.24 | 0.86 | 139.52 |
|  |  | *NDVI, PH, DH* | 0.26 | 0.85 | 140.40 |
|  |  | *NDVI, DH, EARDENS* | 0.24 | 0.86 | 141.87 |
|  |  | *NDVI, PH* | 0.19 | 0.89 | 142.83 |
|  |  | *NDVI, PH, DH, EARDENS* | 0.26 | 0.85 | 143.28 |
|  |  | *DH, EARDENS* | 0.17 | 0.90 | 143.69 |
|  |  | *DH* | 0.12 | 0.93 | 144.53 |
|  |  | *NDVI, PH, EARDENS* | 0.20 | 0.89 | 144.99 |
|  |  | *NDVI* | 0.10 | 0.94 | 145.13 |
|  |  | *NDVI, EARDENS* | 0.13 | 0.93 | 146.31 |
|  |  | *PH, DH, EARDENS* | 0.17 | 0.90 | 146.69 |
|  |  | *EARDENS* | 0.08 | 0.96 | 146.71 |
|  |  | *PH, DH* | 0.12 | 0.93 | 146.79 |
|  |  | *PH, EARDENS* | 0.11 | 0.94 | 147.68 |
|  |  | *PH* | 0.04 | 0.98 | 148.79 |
|  |  |  |  |  |  |
| **Landraces and modern** |  | *PH* | 0.71 | 1.29 | 176.62 |
|  |  | *PH, EARS* | 0.71 | 1.28 | 178.42 |
|  |  | *PH, DH* | 0.71 | 1.28 | 178.68 |
|  |  | *NDVI, PH* | 0.71 | 1.28 | 179.03 |
|  |  | *PH, DH, EARS* | 0.72 | 1.27 | 180.70 |
|  |  | *NDVI, PH, DH* | 0.71 | 1.27 | 180.97 |
|  |  | *NDVI, PH, EARS* | 0.72 | 1.27 | 180.97 |
|  |  | *NDVI, PH, DH, EARS* | 0.72 | 1.27 | 183.18 |
|  |  | *NDVI, DH* | 0.34 | 1.94 | 220.17 |
|  |  | *NDVI, DH, EARS* | 0.35 | 1.93 | 222.44 |
|  |  | *NDVI* | 0.26 | 2.05 | 223.26 |
|  |  | *NDVI, EARS* | 0.27 | 2.04 | 225.64 |
|  |  | *DH* | 0.22 | 2.12 | 226.39 |
|  |  | *DH, EARS* | 0.23 | 2.10 | 228.06 |
|  |  | *EARS* | 0.06 | 2.32 | 235.63 |
|  |  |  |  |  |  |

|  | **2018** | | | |
| --- | --- | --- | --- | --- |
|  |  |  |  |  |
| **Set** | **Parameters** | ***R^2^*** | **RSME** | **BIC** |
|  |  |  |  |  |
| **Landraces** | *PH* | 0.02 | 0.76 | 123.70 |
|  | *NDVI* | 0.01 | 0.78 | 125.61 |
|  | *EARS* | 0.00 | 0.78 | 126.17 |
|  | *DH* | 0.00 | 0.79 | 126.46 |
|  | *NDVI, PH* | 0.04 | 0.77 | 127.04 |
|  | *PH, EARS* | 0.03 | 0.77 | 127.52 |
|  | *PH, DH* | 0.03 | 0.78 | 127.87 |
|  | *NDVI, EARS* | 0.02 | 0.78 | 127.93 |
|  | *NDVI, DH* | 0.01 | 0.78 | 128.59 |
|  | *DH, EARS* | 0.00 | 0.78 | 129.08 |
|  | *NDVI, PH, EARS* | 0.05 | 0.77 | 129.43 |
|  | *NDVI, PH, DH* | 0.04 | 0.77 | 129.95 |
|  | *PH, DH, EARS* | 0.03 | 0.78 | 130.54 |
|  | *NDVI, DH, EARS* | 0.02 | 0.78 | 130.92 |
|  | *NDVI, PH, DH, EARS* | 0.04 | 0.77 | 132.47 |
|  |  |  |  |  |
| **Modern** | *NDVI* | 0.20 | 0.88 | 138.00 |
|  | *NDVI, PH* | 0.23 | 0.86 | 139.25 |
|  | *NDVI, PH* | 0.20 | 0.88 | 140.76 |
|  | *NDVI, EARDENS* | 0.20 | 0.88 | 140.77 |
|  | *NDVI, PH, PH* | 0.23 | 0.86 | 142.03 |
|  | *NDVI, PH, EARDENS* | 0.23 | 0.86 | 142.06 |
|  | *PH* | 0.11 | 0.92 | 143.45 |
|  | *NDVI, PH, EARDENS* | 0.20 | 0.88 | 143.55 |
|  | *PH, PH* | 0.16 | 0.90 | 143.75 |
|  | *PH* | 0.11 | 0.93 | 143.84 |
|  | *NDVI, PH, PH, EARDENS* | 0.23 | 0.86 | 144.98 |
|  | *PH, EARDENS* | 0.11 | 0.93 | 146.47 |
|  | *PH, EARDENS* | 0.10 | 0.93 | 146.84 |
|  | *PH, PH, EARDENS* | 0.15 | 0.90 | 146.86 |
|  | *EARDENS* | 0.01 | 0.98 | 149.29 |
|  |  |  |  |  |
| **Landraces and modern** | *NDVI, PH, PH* | 0.69 | 1.31 | 183.50 |
|  | *NDVI, PH* | 0.66 | 1.36 | 184.62 |
|  | *NDVI, PH, PH, EARDENS* | 0.69 | 1.30 | 186.03 |
|  | *NDVI, PH, EARDENS* | 0.66 | 1.36 | 187.23 |
|  | *PH* | 0.63 | 1.44 | 187.29 |
|  | *PH, PH* | 0.64 | 1.40 | 187.74 |
|  | *PH, EARDENS* | 0.62 | 1.44 | 190.38 |
|  | *PH, PH, EARDENS* | 0.64 | 1.40 | 190.82 |
|  | *NDVI, PH* | 0.47 | 1.70 | 207.42 |
|  | *NDVI, PH, EARDENS* | 0.47 | 1.70 | 210.06 |
|  | *PH* | 0.26 | 2.01 | 221.29 |
|  | *NDVI* | 0.24 | 2.04 | 222.69 |
|  | *PH, EARDENS* | 0.26 | 2.02 | 224.40 |
|  | *NDVI, EARDENS* | 0.25 | 2.04 | 225.41 |
|  | *EARDENS* | 0.00 | 2.36 | 237.31 |
|  |  |  |  |  |
